# Placenta Praevia — a Fatal Case

**Published:** 1873-06-15

**Authors:** A. B. McCandless

**Affiliations:** Columbus City, Iowa


### PLACENTA PRAEVIA.—A FATAL CASE.

BY A. B. M'CANDLESS, M.D., COLUMBUS CITY, IOWA.

*Editors Medical Examiner:*—A case with the above caption came under my notice and professional care which I desire to present to the profession through the medium of your excellent journal.

On the 24th of February, 1873, I was summoned to visit Mrs. Margaret M——. Found my patient suffering from uterine hæmorrhage; seven months advanced in pregnancy; had borne seven children; 44 years of age.

In consequence of excessive tenderness of the labiæ and vulva I desisted from an examination per vagina; but from all information gathered by interrogating the patient I suspected a case of placenta praevia, with partial attachment to the walls of the uterus of right side. I prescribed fluid extract secal. cor. one ounce; sulph. morph. four grains—M. and S.—fifteen drops every three hours, until hæmorrhage is arrested. Prescription seemed to act admirably. Patient in a few days was engaging in light domestic duties. On March 18th I was again summoned to witness the same conditon, only somewhat aggravated. I made same prescription, enjoining rest, and tepid anodyne fomentations over bowels, and she again rallied and again engaged to some extent in household duties, feeling as well as could be expected.

On the morning of the 23d of the present month I was again called to witness the same difficulty. I proceeded to examine per vagina, and my fears and forebodings were fully realized. Found the os dilated and dilatable, and placenta presenting from right side of the uterus. I apprised the husband of the nature and gravity of the case; and in consequence of the heavy responsibility in the case I suggested council, and Dr. B. G. Neal, of Columbus Junction, was brought to my aid.

Although the os was well dilated, there was little or no labor pain. The former prescription, increased a little, seemed to control flooding. In council it was agreed to invite uterine contraction. Her full period was almost accomplished. By increasing the secal. cor. to thirty drops every half-hour, after two doses there was some uterine contraction but not sufficient to accomplish anything in way of labor, but in a measure controlled hæmorrhage. Two more doses of same quantity was given at same interval, with but little effect. I might remark here that same extract had been used on former occasions, with happy and desired results.

It was then deemed proper to rupture membranes, and induce labor if possible in that way, and increase the extract ten grains. Nature then seemed, with the aid of the means just named, to make an effort to expel contents of womb. The head entered the strait, but the effects of nature seemed to fail. The forceps were applied and child extracted. Born 4 o'clock, 14th inst. Patient very much reduced, but seemed to rally by aid of diffusable stimulants. It was now thought desirable to extract the placenta, when it was found to be about one-third firmly attached to right side of uterus. The hand was gently inserted in cavity of womb, and the attachments broken up and the placenta extracted. Womb contracted, with little hæmorrhage. Stimulants were administered liberally and patient rallied a little; but soon she became restless; pulse unsteady and irregular; extremities cold. By use of derivatives and diffusable stimulants, circulation improved a little. Unceasing efforts were made to restore circulation, but without avail. At 12 o'clock, 25th inst., she died.

Mr Editor, I wish to make two or three inquiries, and would be pleased to have your opinion, or that of any of your readers. First, was the course pursued the most scientific? or would it have been better to have turned and delivered at an earlier period? Second, from the fact that there was very little hæmorrhage after the birth of the child and expulsion of the placenta, was death caused by exhaustion, or an overpowering of mere force, or either? Third, would the amount of secal. cor. taken (about four ounces in all) contain sufficient ergotine to produce disorganization of blood? Any ideas on this subject, either by criticism or othewise will be appreciated.

